# Comparison of different automated lesion delineation methods for metabolic tumor volume of ^18^F-FDG PET/CT in patients with stage I lung adenocarcinoma

**DOI:** 10.1097/MD.0000000000009365

**Published:** 2017-12-22

**Authors:** Xiao-Yi Wang, Yan-Feng Zhao, Ying Liu, Yi-kun Yang, Zheng Zhu, Ning Wu

**Affiliations:** a PET/CT Center; b Department of Diagnostic Radiology; c Department of Thoracic Surgery, National Cancer Center/Cancer Hospital, Chinese Academy of Medical Sciences and Peking Union Medical College, Beijing, China.

**Keywords:** adaptive thresholding, delineation method, lung adenocarcinoma, metabolic tumor volume, PET

## Abstract

The aim of the study was to investigate the suitable segmentation method in small, low uptake and heterogeneous nodules of stage I lung adenocarcinoma.

133 stage I lung adenocarcinoma patients with ^18^F-FDG PET/CT scans were enrolled in this retrospective study. All lesions were divided into different groups according to nodule density, nodule size, and the maximum standard uptake value (SUV_max_) level. Four different PET segmentation methods were performed, including percentage threshold of SUV_max_ (T42% and T42% × RC), gradient-based threshold (adaptive iterative algorithm, AT-AIA), and background-related threshold (adaptive thresholding at 40% SUV_max_, AT40%) approaches. The MTVs were evaluated and compared with CT volume (CTV). Percentage volume error (%VE) compared to CTV was calculated and the correlations between MTVs and CTV were analyzed.

AT-AIA had the highest accuracy in large, high uptake, and solid nodules (72.5%, 72.4%, and 65.6%, respectively). AT40% had the highest accuracy in small, low uptake and nonsolid nodules (56.6%, 56.1%, and 62.6%, respectively). In part-solid nodules, the accuracy of AT-AIA (60.0%) and AT40% (56.7%) were higher than that of T42% and T42% × RC. The MTV of AT-AIA was in excellent correlation with the CTV in solid nodules (*R* = 0.831, *P* < .001) and in high uptake nodules (*R* = 0.830, *P* < .001). The MTV of AT40% was in good correlation with the CTV in nonsolid nodules (*R* = 0.686, *P* = .003) and in part-solid nodules (*R* = 0.731, *P* < .001).

AT40% showed best performance in small, low uptake, nonsolid and part-solid lesions. AT-AIA was suitable for large, high uptake, and solid lesions.

## Abstract 2

Supplemental Digital Content is available in the text

## 1 Introduction

Positron emission tomography/ computed tomography with 2-deoxy-2-[^18^F]fluoro-D-glucose (^18^F-FDG PET/CT) shows its usefulness in tumor staging and follow-up. Recently, several researches have already proved that maximum standard uptake value (SUV_max_), metabolic tumor volume (MTV), and total lesion glycolysis (TLG) had prognostic and predictive values in nonsmall cell lung cancer (NSCLC) patients.^[1–5]^ Higher SUV_max_ and adenocarcinoma histology were associated with shorter disease-free survival (DFS).^[6]^ High SUV_max_ and high MTV of the primary tumor are independent prognostic factors of shorter DFS in early stage of NSCLC without lymph node metastasis.^[7,8]^ In addition, SUV_max_, MTV, and TLG have prognostic role on NSCLC patients treated with stereotactic body radiation therapy; only MTV and TLG have a predictive value for DFS when tumors are larger than 3 cm.^[9,10]^

The lesions in those previous studies were generally with solid nodule type as well as high FDG uptake.^[5–8]^ How about the predictive ability in ground-glass opacity nodule (GGN) with low uptake lesions? Goudarzi et al^[11]^ reported that pure bronchioloalveolar carcinoma (BAC) exhibits smaller size, lower uptake, and lower tumor density than invasive adenocacinoma, and many BACs have low SUVs (<2.0). Khalaf et al^[12]^ reported that although the SUV_max_ cutoff value of 2.5 is a useful tool in the evaluation of large pulmonary nodules (>1.0 cm), it has no or minimal value in the evaluation of small pulmonary nodules (≤1.0 cm). Although generally, small GGN with low uptake are going to fall well into the favorable prognostic category, these patients need long-term follow-up exams.

Various automated methods are currently used to segment regions of interest in PET/CT scans, including fixed SUV threshold (e.g., SUV2.5), percentage threshold of SUV_max_ (e.g., T42%), gradient-based threshold (adaptive iterative algorithm, AT-AIA), and background-related threshold (AT40%) approaches. However, up to now, it is still challenging to define MTV accurately for heterogeneous and low uptake lung nodules and prone to inter- and intraobserver variability. It is known that a single threshold SUV method is not universally applicable to all clinical scenarios,^[13,14]^ especially in low FDG uptake lesions. The fixed threshold method was not used in our study since it ignores the background. Currently, the percentage threshold (T42%) method is widely used in lung cancer, which is based on homogenous phantom study with high contrast, so as to more applicable to the solid nodules larger than 20 mm and with high FDG uptake.^[7,15]^ Percentage threshold can be performed rapidly and consistently, with less inter-observer variability. The adaptive iterative delineation method (AT-AIA) is more advanced and complex, which uses an iterative algorithm to find a threshold value that separates the tumor from the background tissue by weighting SUV_max_ and SUV_mean_ within the bounding box. AT-AIA was usually used on solid nodules larger than 20 mm with high FDG uptake.^[16,17]^ But there is not enough clinical evidence that it is suitable for small and low uptake lesions. The AT-AIA method tends to find the largest gradient at the border of the lesions, but in low uptake lesions, the gradient is relatively low. So, this method may not be a good choice in such a clinical scenario. The AT40% method, considering the metabolic contrast between lesion and background uptake and the location of the lesion, may improve the segment accuracy in small and low uptake lesions, although with the manual background region of interest procedure, which may introduce more inter-observer variability than the other methods.^[18]^ Firouzian et al^[18]^ found that these automated lesion delineation methods have high variation in small lesions. The aim of this paper is to investigate the suitable segmentation method in small, low uptake and heterogeneous nodules of stage I lung adenocarcinoma.

## 2 Materials and methods

### 2.1 Subjects

A total of 133 patients with stage I adenocarcinoma who performed ^18^F-FDG PET/CT scans prior to surgery in our hospital from June 2005 to June 2012 were enrolled in this retrospective study. The informed consent was waived because of the retrospective nature of this study. This was agreed by the local ethics committee and approval from the ethics committee was granted. There were 65 males and 68 females with age ranged 35 to 84 years (mean 60 years). The locations of the lung nodules were as follow: 33 lesions in the left upper lobe, 24 lesions in the left lower lobe, 47 lesions in the right upper lobe, 7 lesions in the right middle lobe, and 22 lesions in the right lower lobe.

All lesions were viewed and judged by 3 experienced radiologists, with 14 years, 17 years, and 30 years working experience respectively. The measurements were performed by 2 senior radiologists, and the time separation between each measurement was 4 weeks. The radiologists were blinded to each other's definition of the lesions. The disagreement was decided by discussion. According to nodule density, the lesions were divided into 3 types: nonsolid, part-solid, and solid. Nonsolid nodule which means pure GGN, defined as an area of hazy increased attenuation that does not obscure underlying bronchial structures or vascular margins on high-resolution computed tomography (HRCT).^[19]^ Part-solid nodule which means mixed GGN, defined as mixed nonsolid and solid components.^[19–21]^ Since the long diameter of 2 cm was the cut-off value of T1a with T1b lung cancer, according to nodule size, the lesions were divided into 2 groups: small lesions (long diameter≤20 mm) and large lesions (long diameter>20 mm). Although the SUV_max_ threshold of 2.5 is generally chosen to maximize sensitivity of malignancy detection, the FDG uptake in early stage lung adenocarcinoma was lower than other lung cancer.^[22,23]^ Therefore, in this study, we considered SUV < 2.0 as low uptake lesion, SUV > 2.0 as high uptake lesion. According to FDG uptakes, the lesions were divided into 2 groups: low uptake lesions (SUV_max_ ≤2) and high uptake lesions (SUV_max_ >2).

### 2.2 ^18^F- FDG PET/CT study

^18^F-FDG PET/CT was performed using an integrated PET/CT (Discovery ST, GE Healthcare). All patients in this study were scanned on the same PET/CT machine. Patients’ blood glucose was between 120 and 200 mg/dL before undergoing PET/CT examination. Patients received 3.70 to 4.44 MBq/kg of ^18^F-FDG intravenously, followed by a whole body PET/CT scan 60 to 70 minutes later. The PET images were obtained with 3 min acquisition per bed position, with slice thickness of 3.27 mm. Scan from skull vertex to upper-thigh resulted in an acquisition time of 18 to 21 minutes. All PET images were reconstructed using an iterative algorithm (ordered-subset expectation maximization, OSEM) with CT-based attenuation correction. Spiral CT was performed with a tube voltage of 120 kV, tube current of 150 mA, 3.75 mm slice thickness and 3.75 mm interval, at 0.8 s per rotation. The attenuation correction scan was performed from vertex to upper thighs and no contrast was used for this examination. Breathing-hold chest CT without contrast was performed then, with a tube voltage of 120 kV, tube current of 205 mA, slice thickness of 5 mm and 1.25 mm, with 5 mm and 0.8 mm interval respectively, at 0.8 seconds per rotation.

### 2.3 Automated PET delineation methods

Four different automated PET delineation methods were evaluated and compared (Table 1). All segmentation algorithms were implemented on the same software platform in AW 4.6 workstation (Advantage Workstation, GE Healthcare) to optimize workflow and minimize reproducibility drawbacks.

**Table 1:**
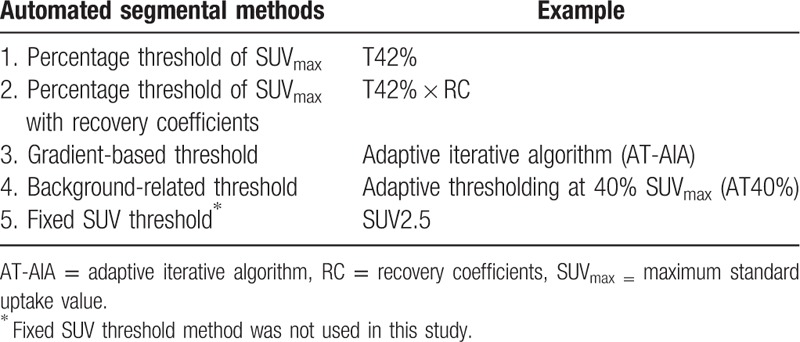
Currently used automated segmental methods.

The first method was thresholding at 42% SUV_max_ (T42%): delineates all voxels with SUVs above or equal to 42% of the maximum SUV inside the selected volume of interest (VOI).

The second method was thresholding at 42% SUV_max_ with recovery coefficients (T42% × RC). This was only done on the lesions whose diameter was less than 30 mm (lesion>30 mm, RC = 1).

As reported earlier,^[24,25]^ the partial volume effect (PVE) is a physical limitation resulting from the poor spatial resolution of PET systems (4–5 mm) which strongly affects the accuracy of the estimation of radioactivity concentration within structures less than 2 or 3 times of the PET spatial resolution. Among all PVE correction methods, more common ones are based on multiplicative numerical factors, recovering the local radioactivity concentration within any small structure which uptakes ^18^F-FDG.^[26,27]^ Recovery Coefficients (RC) was obtained as a function of PET resulting from the threshold-isocontour technique. 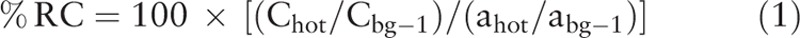 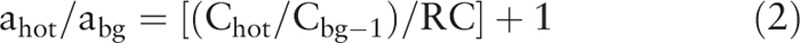


where C_hot_ and C_bg_ are the average counts measured in the hot sphere ROIs and the average counts in all background ROIs, respectively, whereas a_hot_/a_bg_ is the ratio of the true radioactivity concentration in the hot sphere and in the background. Gallivanone et al^[26]^ have reported the method for PVE correction of oncological lesions in clinical studies, based on RC and on PET measurements of lesion to background ratio and of lesion metabolic volume. The validation of the PVE correction method resulted to be accurate (>89%) in clinical realistic conditions for lesion diameter > 1 cm, recovering >76% of radioactivity for lesion diameter < 1 cm. Results from patient studies showed that the proposed PVE correction method is suitable and feasible and has an impact on a clinical environment.^[26,28]^ In this study, the RC was derived from PET experimental measurements of small radioactive objects in a priori known object-to-background radioactivity concentration ratio. It came from the work of our previous colleagues^[28]^ (Table 2).

**Table 2:**
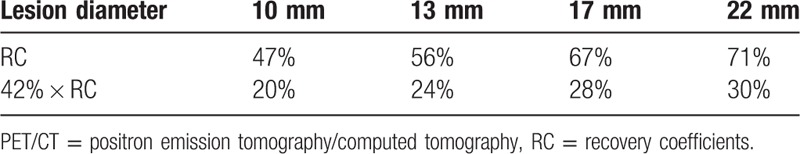
Recovery coefficients (RC) of discovery ST PET/CT.

**Figure 1:**
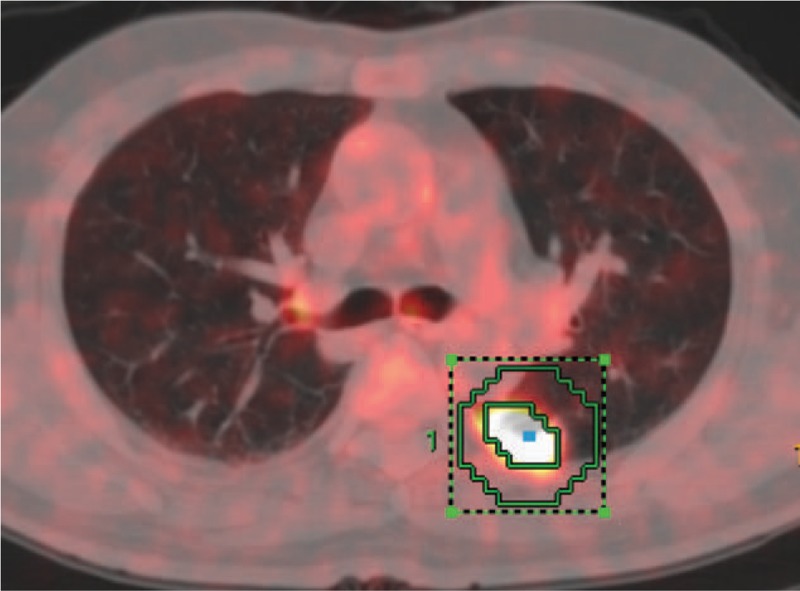
The metabolic tumor volume (MTV) was segmented using an adaptive iterative algorithm (AT-AIA) in PETVCAR. AT-AIA = adaptive iterative algorithm, MTV = metabolic tumor volume, PETVCAR = PET volume computerized assisted reporting.

The third method was adaptive iterative volume delineation by PET Volume Computerized Assisted Reporting software (PETVCAR, GE Healthcare). The PET and CT co-registration was first assessed once the images were loaded into the PETVCAR software. The primary lung cancer PET gray scale and PET/CT fused images were then reviewed in the axial, sagittal, and coronal planes. A boundary box was placed over the image, which was to auto-contour and segment the region of interest, reviewed and adjusted to ensure this 3-dimensional cube contained all the ^18^F-FDG PET positive area and excluded the negative normal tissue. This process was repeated until each ^18^F-FDG PET/CT positive region has been selected and optimized. The lesion metabolic volume was then automatically segmented using an adaptive iterative algorithm (AT-AIA) in PETVCAR which separated the target volume from the background tissue by weighting the SUV_max_ and the SUV_mean_ within the target volume with a weighting factor, represented as a Boolean variable. This weighting factor was automatically set at 0.5^[16]^ (Fig. 1).

And the fourth method was adaptive thresholding at 40% SUV_max_ (AT40%),^[18]^ which adapts the threshold value inside the selected VOI relative to mean background (BG) SUV, calculating T value as thresholding: 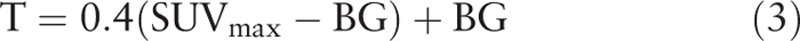


This delineation method required information of background uptake. The background region needs to be defined by the user which might introduce some variations in the results. The background of lung is heterogeneous; mean background SUV has discrepancy at different regions (apex, central, and peripheral region). The user needs to copy the ROI and select the same location at contralateral lung.

### 2.4 Computed tomography volume

**Figure 2:**
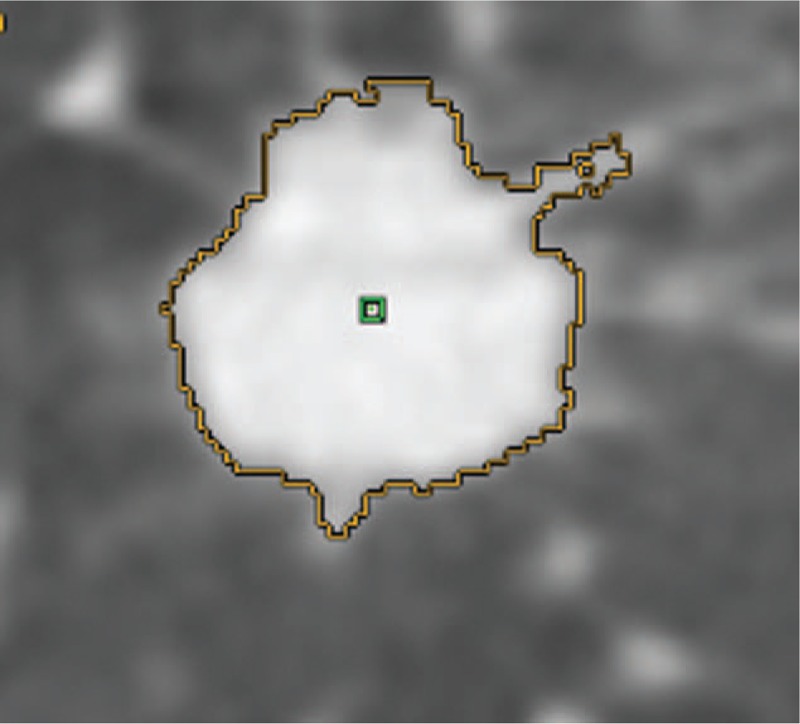
The lesion computed tomography volume (CTV) was measured through Lung VCAR software. CTV = computed tomography volume, VCAR = Lung volume computerized assisted reporting.

When lesion density is different from the density of the surrounding tissues, a computed tomography study in the region of interest can provide lesion anatomical volume (computed tomography volume, CTV). CTV was measured through Lung Volume Computerized Assisted Reporting software (Lung VCAR, GE Healthcare) on the 1.25 mm slice thickness images. Lung VCAR is an image analysis software package for Advantage Workstation systems that uses GE's Volume Viewer software. The analysis mode was used which offers a combination of 2D reformatted views with correlated volume rendering views. In this mode, the software zooms on the volume of interest, automatically calculates the volume of the suspicious spot, and displays the calculated volume on the views. Also, depending on the protocol chosen (nodule consistency and circumscribed situation), it displays the consistency of the detected nodules. The actual volume was measured using an automatic nodule sizing algorithm. Upon entering the analysis mode, the software automatically performs the following operations: step 1- definition of a VOI around the nodule; step 2- determination of nodule consistency (solid, part-solid or nonsolid); step 3- determination of nodule circumscribed situation (well circumscribed, vascularized or juxta-pleural). Then the software automatically computes the segmentation: type 1- if the nodule is well circumscribed, the system calculates its volume and displays it on the views; type 2- if the nodule is vascularized, the system proceeds to an automatic vascular tree extraction, followed by a vessel cut, before calculating and displaying its volume on the views; type 3- if the nodule is juxta-pleural, the system separates it from the pleural wall, before calculating and displaying its volume on the views (Fig. 2).

Reproducibility evaluation was achieved for the implementation by repeating the delineation procedure several times in each patient. The inter-observer variability on the delineation process was less prone to happen because the boundary box was auto-contour and segments the region of interest. The user only need to review and adjust to ensure the 3D-box contained all the FDG positive area and excluded the negative normal tissue. Then the lesion metabolic volume was automatically segmented from the different algorithms. The Intraclass Correlation Coefficient (ICC) was used to estimate the reliability between observers when using the fourth delineation method (AT40%).

The follow up of these patients for progression-free survival (PFS) was performed to further validate whether the delineation method classification reasonable.

### 2.5 Validation and statistics

Percentage volume error (%VE) was calculated using CTV as reference: 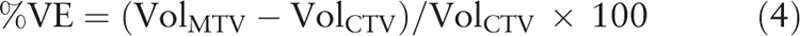


The Vol_MTV_ was the volume of delineated lesions in PET images and Vol_CTV_ was the volume of the delineated lesions in CT images. The discrepancies between the imaging modalities of CT and PET in tumor volume delineation had been reported in previous studies. A difference less than 30% between CTV and MTV was considered clinical acceptable.^[29–34]^ In this study, a difference between ± 50% was considered acceptable, because the lesions in our study were smaller, lower FDG uptake and more heterogeneous than other researches. The accuracy of each method was defined by the percentage of cases which fell within this range. The %VE more than 50% meant overestimated, less than –50% meant underestimated.

The results were evaluated by standard methods including combined t test and Chi-square test. The descriptive data are expressed as the means ± standard deviations. T test was used to analyze the continuous variables, and the chi-square test to compare the categorical variables between groups. The correlation of various MTVs from different segmentation algorithms with CTV was analyzed and evaluated by Pearson correlation. The ordinal data correlation test was performed using the Spearman test. The correlation coefficient (*R* value) = 0.21–0.40 for the poor consistency, *R* value = 0.41–0.60 for the moderate consistency, *R* value = 0.61–0.80 for the good consistency, *R* value = 0.81–1.00 for the excellent consistency.^[35]^

The intraclass correlation coefficient (ICC) was used to estimate the reliability between observers when using the fourth delineation method (AT40%). ICC < 0.40 is for the poor reliability, ICC > 0.75 for the good reliability. Progression-free survival (PFS) was compared by employing the Kaplan–Meier method and Cox proportional-hazard model. *P* *<* .05 were assumed to indicate significant differences. The data were analyzed by SPSS 13.0 software (Chicago).

## 3 Results

According to the classification of lung nodule types analyzed by 3 experienced radiologists, there were 16 nonsolid nodules, 30 part-solid nodules, and 87 solid nodules in all 133 lesions. The SUV_max_, diameter, and CTV of 3 lung nodule types were shown in Table 3. There was statistical significance of SUV_max_ between solid and part-solid nodule, solid and nonsolid nodule, part-solid and nonsolid nodule (*t* = 4.706, *P* < .001; *t* = 4.539, *P* < .001; *t* = 3.269, *P* = .002, respectively). But the diameter and CTV had no statistical significance among the 3 types (all *P*>.05). The nodule types had good consistency with SUV_max,_ (*R* = 0.680, *P* < .001) but not with diameter and CTV (Spearman test).

**Table 3:**
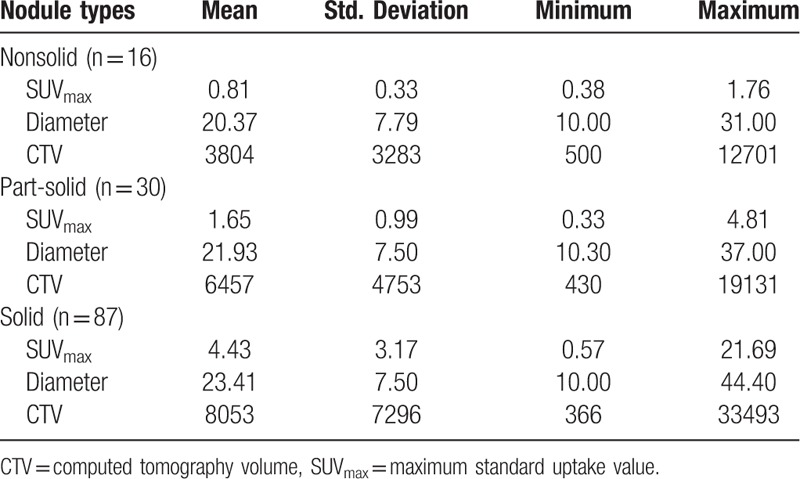
SUV_max_, diameter (mm), and CTV (mm^3^) of 3 lung nodule types.

**Figure 3:**
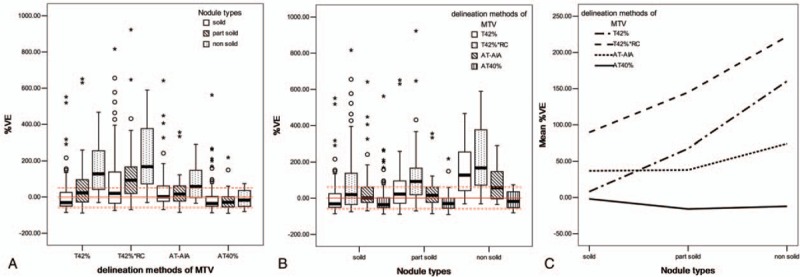
%VE of 4 delineation methods of MTV (A), %VE of 3 nodule types (B), mean %VE of 3 nodule types (C). (A) For each method a group of 3 boxplots are presented, each belonging to lung nodule types. Each boxplot represents the distribution (mean and quartiles) of validation results. (B) For each nodule type, a group of 4 boxplots are presented, each belonging to delineation methods. (C) Mean %VE values are presented with respect to nodule types for 4 methods. %VE = percentage volume error, MTV = metabolic tumor volume.

The MTV, %VE, and SUV_mean_ of 4 automated PET delineation methods were shown in Table 4. The comparisons of VE% (*t*, *P* value) of different groups were shown in Supplemental Digital Content Tables 1, 2, 3, and 4. There was statistical significance between most of each 2 methods of %VE. Figure 3A and B showed the variation of %VE of each delineation method in the 3 types of nodules. The variation of VE% in nonsolid nodule is much larger than that in part-solid nodule and solid nodule. The mean variation of %VE of AT40% is the smallest in the 4 methods. Figure 3C showed that T42% is good at solid nodule, but unstable in part-solid and nonsolid nodule. AT40% and AT-AIA were more stable than the other 2 methods.

**Table 4:**
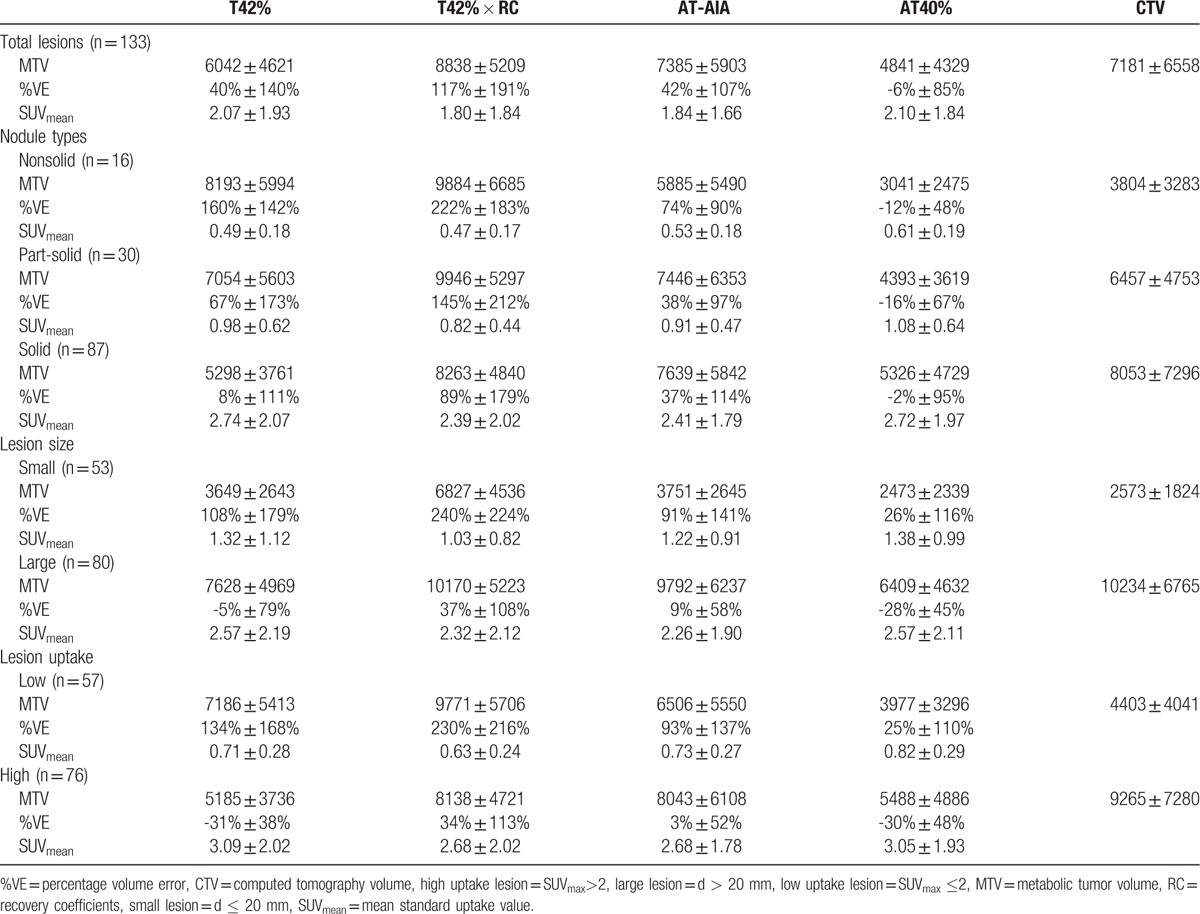
MTV (mm^3^), %VE, and SUV_mean_ of each automated PET delineation methods.

**Figure 4:**
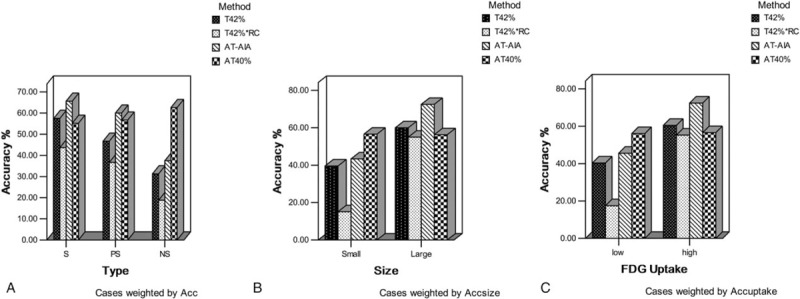
The segmental accuracy of 4 methods in different nodule type (A), size (B), and FDG uptake groups (C). The AT40% method was superior for small, low uptake, nonsolid lesions; the AT-AIA method was superior for large, high uptake, solid lesions. AT-AIA = adaptive iterative algorithm.

According to the criteria that a difference less than ±50% was considered accurate in this study, the segmental accuracy of 4 methods in different nodule type, size and FDG uptake groups were shown in Figure 4. Figure 4 demonstrated that the AT40% method was superior for small, low uptake, nonsolid lesions; the AT-AIA method was superior for large, high uptake, solid lesions. The underestimated and overestimated percentages of 4 methods with variation are shown in Table 5, which showed that AT40% underestimated the lesions in all 3 nodule types compared with the other 3 methods, whereas T42% and T42% × RC usually overestimated the nonsolid and part-solid lesions. However, the comparison of accuracy percentage of 4 methods in different groups had no statistical significance (all *P* *>* .05).

**Table 5:**
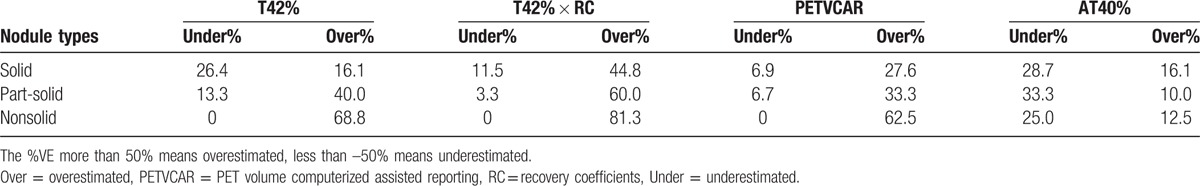
The underestimated and overestimated percentage of 4 methods with variation.

The MTV of AT-AIA had excellent consistency with CTV in solid nodules (*R* = 0.831, *P* < .001) and also in high uptake nodules (*R* = 0.830, *P* < .001). The MTV of AT40% was in good correlation with the CTV in nonsolid nodules (*R* = 0.686, *P* = .003) and in part-solid nodules (*R* = 0.731, *P* < .001). The *R* values of AT-AIA and AT40% were higher than that of T42% in most groups. The *R* value of T42% × RC was not as good as those of the other methods (Supplemental Digital Content Table 5).

The ICC was 0.933 between observers when using the fourth delineation method (AT40%), which means good reliability.

In the survival analysis, we used adaptive iterative algorithm (AT-AIA) in solid lesions, adaptive thresholding (AT40%) in nonsolid and part-solid lesions. In univariate analysis, MTV was significantly associated with PFS (*P* = .04); patients with high MTV were associated with poor prognosis. In multivariate analysis, only MTV was independent prognostic factors among 5 PET/CT metabolic parameters with a *P* value of .031 (RR, 1.118; 95% CI, 1.010–1.237).

**Figure 5:**
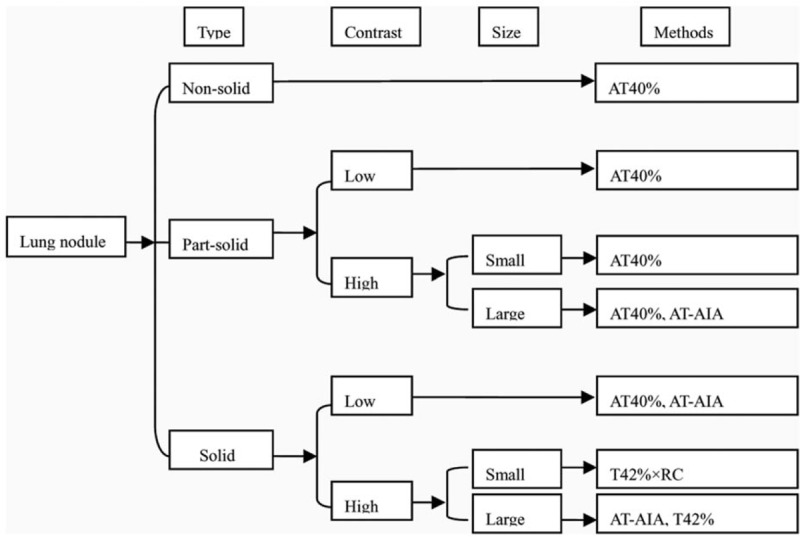
Flow chart summarizing methods’ performance in stage I lung adenocarcinoma. The options for each parameter are presented in rectangles and the best performing method this type of data is presented alongside these rectangles. The methods are ordered according to the corresponding %VE ± SD, accuracy, and the correlation coefficient. %VE = percentage volume error, SD = standard deviation.

The flowchart of summarizing the methods’ performance based on different parameters of 133 lung nodules data is shown in Figure 5. This diagram provides an overview of the relative performance of the best performing methods for different situations. Depending on the types of data and application, clinicians can use this flowchart to aid their selection of the most appropriate method.

## 4 Discussion

In general, early stage lung adenocarcimomas are of lower FDG uptake compared to other histological subtypes of NSCLC, especially the nonsolid and some subtype of part-solid adenocarcimomas.^[11,12,22,23,36–39]^ Smaller nodules especially are more likely to have partial volume effects. The investigation of the segmentation method on small heterogeneous lung nodules with low FDG uptake is still rare. Currently, there is no universally accepted segmentation method for such lesion yet. The aim of this paper is to investigate the suitable segmentation method for small, low uptake and heterogeneous lung cancer lesions.

During the comparison of the MTVs, the “true” volume of the lesions needs to be determined. However, there is no appropriate reference for the evaluation of volumes. Although a number of recent papers use macroscopic specimen obtained from histology as reference,^[40,41]^ there is still problematic since the irregular contraction can occur during tissue fixation, and the criterion of contraction rate is quite different. In Schaefer et al's^[42]^ research, they used pathology as the ground truth or CT as a ground truth surrogate, and recommended consensus contours from multiple PET segmentations as a new reference. Nestle et al^[17]^ calculated “expanded” CT volumes according to the smallest margins recommended for motion correction as the standard (the expansion was 0.15 cm lateral, 0.2 cm anteroposterior, and 0.3 cm craniocaudal), and she thought the expanded CTV appeared to be closest to the true PET volumes. Caldwell et al^[43]^ had also reported that the volumes of chest tumors as measured by PET would be equal or larger than the volumes measured by CT. Previous literatures reported that a difference less than 30% between CTV and MTV was considered clinical acceptable.^[29–34]^ In our retrospective study, the previous recorded and described of each specimen slice to estimate the pathological volume was unavailable. Considering all above reasons, we compared MTVs with CTV and evaluated the accuracy using ±50% as criteria, since the lesions of stage I lung adenocarcinoma in this study were smaller, with lower FDG uptake and more heterogeneity than other researches.

The nonsolid and part-solid lung nodules, in our study, were usually with low FDG uptake (*R* = 0.68). It means with the increase of nodule density from GGN to solid nodule, the FDG uptake increased. Our study showed that the delineated MTV were overestimated in most of the cases using T42% in nonsolid and part-solid lung nodules. Moreover, the threshold of T42% × RC is too low, so as to involve more false positive background uptakes in nonsolid and part-solid nodules. AT40% seems, therefore, the best segmentation method in low FDG uptake nodules. It adapts the threshold value according to the mean background SUV. The uptake in normal lung tissue is heterogeneous which might introduce some variations. AT40% is the only method in this study considering the metabolic contrast between target lesion and background uptake information, and considering the location of the lesion at different region of the lung which may improve the accuracy. It appears to be more stable against the heterogeneity of tumor uptake and the broad variation of SUV_max_ values than the other methods in this study.^[17]^ Therefore AT40% should be the optimal choice in nonsolid and part-solid nodules with low uptake. Moreover, in the reproducibility evaluation, there was good reliability between observers when using AT40% method. Furthermore, in the survival analysis, using AT40% method was potentially validated reasonable.

Both AT-AIA and T42% showed good performance in large, high uptake solid lesions in our study. However, AT-AIA seemed the best method. It showed the highest correlation value with CTV, and the highest accuracy in this study.

T42% is used widespread clinically. This method is based on the homogeneous phantom study with high contrast (8:1). It is usually applicable to tumors whose diameters are larger than 20 mm and with high uptake.^[17]^ It is known that due to the physical principles and the physical limitation resulting from the poor spatial resolution of PET systems, T42% is not applicable to low uptake small lesion.^[44]^ Messa et al^[29]^ and Bradley et al^[31]^ reported the discrepancies between the imaging modalities of PET and CT in tumor volume delineation. When correlated with CTV, PET either underestimated or overestimated the volume due to a number of factors especially partial volume effect.^[45]^ In this study, T42% and T42% × RC overestimated the MTV of nonsolid nodules in almost all of the cases. In these cases, the partial volume effect affects the accurate estimation of FDG uptake strongly. Another reason might be that delineation volumes include the noise of background or nontumor tissues since the contrast between lesion and background is too small to detect.

Firouzian et al^[18]^ reported that lesion size and contrast had impact on the relative performance of the delineation methods. In this study, we considered that lesion type was another important impact factor in addition to lesion size and contrast. The lesion type had good correlation with SUV_max_ in this study. Analyzing nodule type is more straightforward than measuring the SUV_max_ and diameter. Therefore, the radiologist should firstly consider about lung nodule type before selecting delineation method. The survival analysis of these patients was potentially validated that the delineation method classification according to the nodule type and FDG uptakes is reasonable.

The limitations of this study are as follows. The first limitation is the lack of correlation with pathological specimens, so the true representation of the tumor volume is not known. But the correlation of imaging with pathological specimens is problematic because of the contraction that can occur during tissue fixation. Xu et al^[46]^ reported that although the change ratio of the sample dimensions before and after fixation was considered, manual measurement errors could not be avoided, and they failed to overlap the volumes from PET imaging and histopathology because of the lack of reliable markers in pathologic sections. Instead of comparing with pathological specimens, we evaluate the agreement between MTV and CTV using ± 50% as criteria. However, it is also not absolute certainty which needs to be carried out on more future studies. Second, the insufficient numbers of nonsolid and part-solid nodules and the heterogeneous distribution in 3 nodule types leaded to the statistical significance could not be demonstrated. The AT40% method was not strikingly better overall, yet is was superior for small, low uptake, nonsolid lesions. However, the number of these lesions was relatively small. For the future research, it would be helpful to include more nodules, especially nonsolid and part-solid ones, to improve statistical validity. Third, we did not investigate the impact caused by the different proportion of solid components within the part-solid nodules.

## 5 Conclusions

Lesion type, nodular size, and FDG uptake had big impact on the relative performance of the delineation methods. AT40% showed best performance in small, low uptake, nonsolid and part-solid lesions. AT-AIA was suitable for relatively large, high uptake, solid lesions.

## Supplementary Material

Supplemental Digital Content
