# Supplementary material for: Comparison of different automated lesion delineation methods for metabolic tumor volume of 18F-FDG PET/CT in patients with stage I lung adenocarcinoma
Source: Medicine (Baltimore). 2017 Dec 22;96(51):e9365. doi: 10.1097/MD.0000000000009365 (PMC5758229; doi:10.1097/MD.0000000000009365)
Supplement: Supplemental Digital Content [file medi-96-e9365-s001.doc]

| **Supplemental Digital Content Table 1** The comparison of VE% of four methods in all 133 lung nodules | | |
| --- | --- | --- |
| Method 1 | Method 2 | *P* |
| T42% | T42%×RC | 0.000 |
| T42% | AT-AIA | 0.824 |
| T42% | AT40% | 0.000 |
| T42%×RC | AT-AIA | 0.000 |
| T42%×RC | AT40% | 0.000 |
| AT-AIA | AT40% | 0.000 |

| **Supplemental Digital Content Table 2** The comparison of VE% of four methods in three nodule types according to lesion density | | | |
| --- | --- | --- | --- |
|  | Method 1 | Method 2 | *P* |
| Solid nodule | T42% | T42%×RC | 0.000 |
|  | T42% | AT-AIA | 0.000 |
|  | T42% | AT40% | 0.134 |
|  | T42%×RC | AT-AIA | 0.000 |
|  | T42%×RC | AT40% | 0.000 |
|  | AT-AIA | AT40% | 0.000 |
| Part-solid nodule | T42% | T42%×RC | 0.000 |
|  | T42% | AT-AIA | 0.272 |
|  | T42% | AT40% | 0.001 |
|  | T42%×RC | AT-AIA | 0.001 |
|  | T42%×RC | AT40% | 0.000 |
|  | AT-AIA | AT40% | 0.001 |
| Non-solid nodule | T42% | T42%×RC | 0.012 |
|  | T42% | AT-AIA | 0.013 |
|  | T42% | AT40% | 0.000 |
|  | T42%×RC | AT-AIA | 0.002 |
|  | T42%×RC | AT40% | 0.000 |
|  | AT-AIA | AT40% | 0.000 |

| **Supplemental Digital Content Table 3** The comparison of VE% of four methods in two groups according to lesion size | | | |
| --- | --- | --- | --- |
|  | Method 1 | Method 2 | *P* |
| Small nodule | T42% | T42%×RC | 0.000 |
|  | T42% | AT-AIA | 0.381 |
|  | T42% | AT40% | 0.000 |
|  | T42%×RC | AT-AIA | 0.000 |
|  | T42%×RC | AT40% | 0.000 |
|  | AT-AIA | AT40% | 0.000 |
| Large nodule | T42% | T42%×RC | 0.000 |
|  | T42% | AT-AIA | 0.045 |
|  | T42% | AT40% | 0.004 |
|  | T42%×RC | AT-AIA | 0.002 |
|  | T42%×RC | AT40% | 0.000 |
|  | AT-AIA | AT40% | 0.000 |

| **Supplemental Digital Content Table 4** The comparison of VE% of four methods in two groups according to lesion uptake | | | |
| --- | --- | --- | --- |
|  | Method 1 | Method 2 | *P* |
| Low uptake nodule | T42% | T42%×RC | 0.000 |
|  | T42% | AT-AIA | 0.029 |
|  | T42% | AT40% | 0.000 |
|  | T42%×RC | AT-AIA | 0.000 |
|  | T42%×RC | AT40% | 0.000 |
|  | AT-AIA | AT40% | 0.000 |
| High uptake nodule | T42% | T42%×RC | 0.000 |
|  | T42% | AT-AIA | 0.000 |
|  | T42% | AT40% | 0.762 |
|  | T42%×RC | AT-AIA | 0.000 |
|  | T42%×RC | AT40% | 0.000 |
|  | AT-AIA | AT40% | 0.000 |

| **Supplemental Digital Content Table 5** The correlation coefficient (R value) of four MTVs with CTV | | | | | | | | |
| --- | --- | --- | --- | --- | --- | --- | --- | --- |
|  | MTV_T42%_ | | MTV_T42%×RC_ | | MTV_PETVCAR_ | | MTV_AT40%_ | |
|  | R value | *P* | R value | *P* | R value | *P* | R value | *P* |
| **Total lesions** | 0.522 | 0.000 | 0.370 | 0.000 | 0.776 | 0.000 | 0.714 | 0.000 |
| **Nodule types** |  |  |  |  |  |  |  |  |
| Solid | 0.678 | 0.000 | 0.373 | 0.000 | 0.831 | 0.000 | 0.701 | 0.000 |
| Part-solid | 0.607 | 0.000 | 0.614 | 0.000 | 0.728 | 0.000 | 0.731 | 0.000 |
| Non-solid | 0.718 | 0.002 | 0.630 | 0.009 | 0.645 | 0.007 | 0.686 | 0.003 |
| **Lesion size** |  |  |  |  |  |  |  |  |
| Small | 0.297 | 0.031 | 0.564 | 0.000 | 0.572 | 0.000 | 0.565 | 0.000 |
| Large | 0.392 | 0.000 | 0.217 | 0.053 | 0.702 | 0.000 | 0.640 | 0.000 |
| **Lesion contrast** |  |  |  |  |  |  |  |  |
| Low | 0.698 | 0.000 | 0.646 | 0.000 | 0.753 | 0.000 | 0.727 | 0.000 |
| High | 0.759 | 0.000 | 0.422 | 0.000 | 0.830 | 0.000 | 0.708 | 0.000 |
| MTV: metabolic tumor volume, CTV: computed tomography volume | | | | | | | | |
